# Use of Soy-Based Formulas and Cow's Milk Allergy: Lights and Shadows

**DOI:** 10.3389/fped.2020.591988

**Published:** 2020-11-17

**Authors:** Elvira Verduci, Elisabetta Di Profio, Lucia Cerrato, Giulia Nuzzi, Luca Riva, Giulia Vizzari, Enza D'Auria, Maria Lorella Giannì, Gianvincenzo Zuccotti, Diego G. Peroni

**Affiliations:** ^1^Department of Health Sciences, University of Milan, Milan, Italy; ^2^Department of Pediatrics, Vittore Buzzi Children's Hospital, University of Milan, Milan, Italy; ^3^Section of Paediatrics, Department of Clinical and Experimental Medicine, University of Pisa, Pisa, Italy; ^4^Fondazione IRCCS Ca' Granda Ospedale Maggiore Policlinico, NICU, Milan, Italy; ^5^Department of Clinical Science and Community Health, University of Milan, Milan, Italy

**Keywords:** soy-based formula, infant nutrition, cow's milk allergy, vegetables beverages, soybean, nutritional status

## Abstract

Soybean (*Glycine max*) is a species of legume native to East Asia and used in childhood diet for over 2,000 years in the East. Soy protein formulas have been available for almost a century. Nowadays, the increase in cow's milk allergy and vegetarian dietary preferences are driving consumers toward cow's milk alternatives. In this paper, we reviewed the nutritional composition of soy-based infant formula and discussed their possible use in pediatric age, mainly focusing on prevention and treatment of cow's milk allergy. Protein quality is determined by digestibility and amino acid content. Purified or concentrated vegetable proteins (e.g., soy protein and gluten) have high digestibility (>95%), similar to those of animal ones. For some intact vegetable products (e.g., whole cereals and pulses), protein digestibility is lower (80–90%). Food processing and heat treatment also influence protein digestibility. Considering these data, we tried to evaluate the possible use of soybean and derivatives in pediatric age, including the nutritional composition of soy formulas and the clinical indications for their use. Moreover, since plant-based beverages are being perceived as healthy by consumers and their use is growing on the market, we recommend that soy drink should not be used as a substitute for infant formulas or cow's milk in children younger than 24 months.

## Introduction

The soybean (*Glycine max* L.) is a legume crop of East Asian origin, but its use has nowadays spread worldwide due to its nutritional value (i.e., high protein and oil contents). In the East, soybeans are used to produce traditional foods such as miso, tofu, natto, tempeh, soymilk, soy sauce, and soy paste. Conversely, in the West, soybeans are mainly processed to obtain full-fat flakes that are then defatted by using organic solvents and pressed into soybean meal, a high-quality protein source. This is subsequently used to obtain texturized vegetable protein, soy concentrate, and soy isolates, used as a protein supplement for various foods, including infant formulas (1, 2).

In the present paper, we reviewed the nutritional composition of soy-based infant formula (SIF) and discussed their possible use in pediatric age, mainly focusing on prevention and treatment of cow's milk allergy (CMA). This paper could be very useful for all health-care professionals (especially pediatricians and nutritionists) who deal with giving practical advice to families, based on scientific evidence.

## Nutrient Content of Soybean

**Figure 1:**
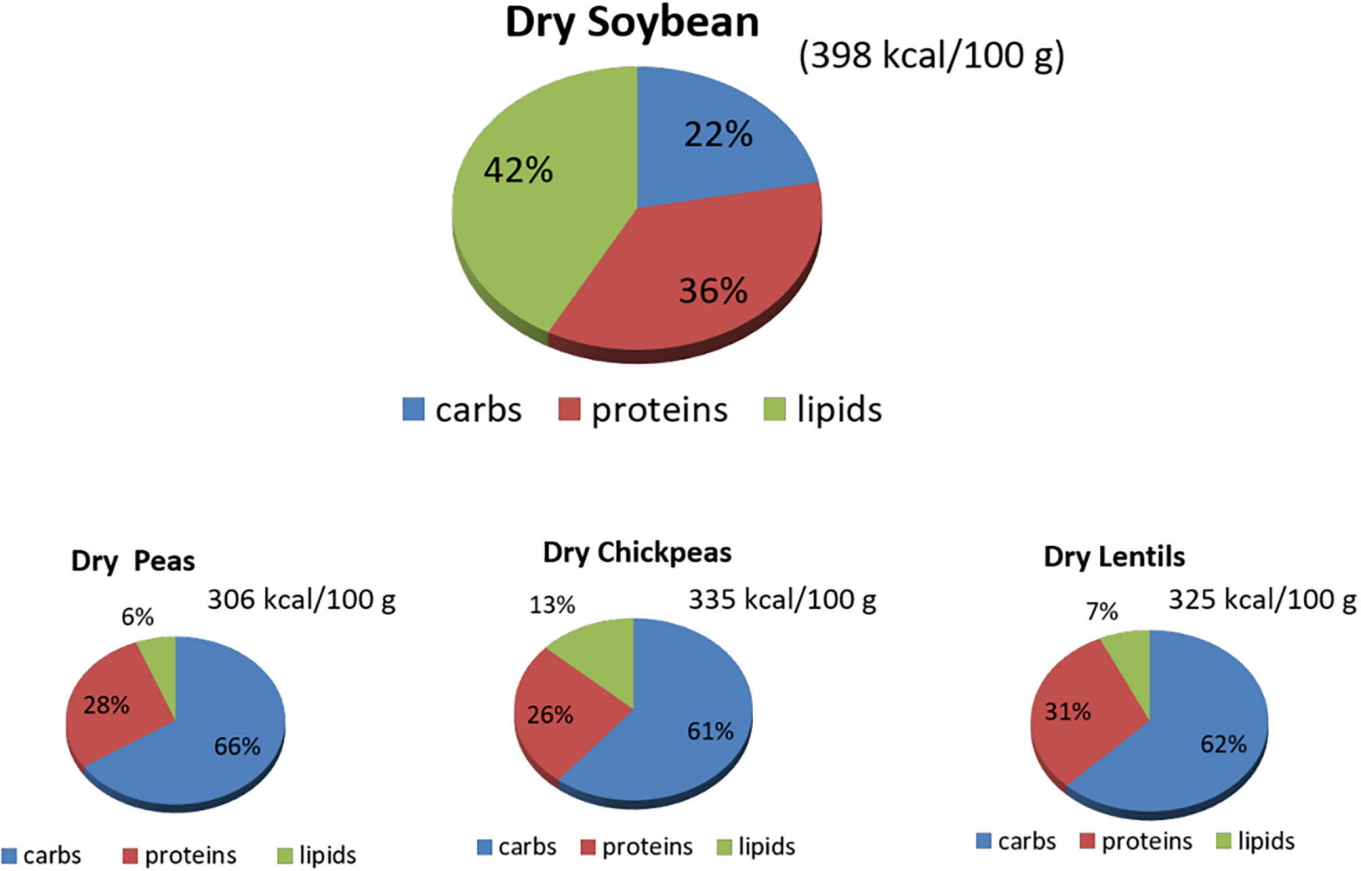
Macronutrients' content (%) in raw dry soybeans, peas, chickpeas, and lentils. Source: reference (8). carbs, carbohydrates.

**Figure 2:**
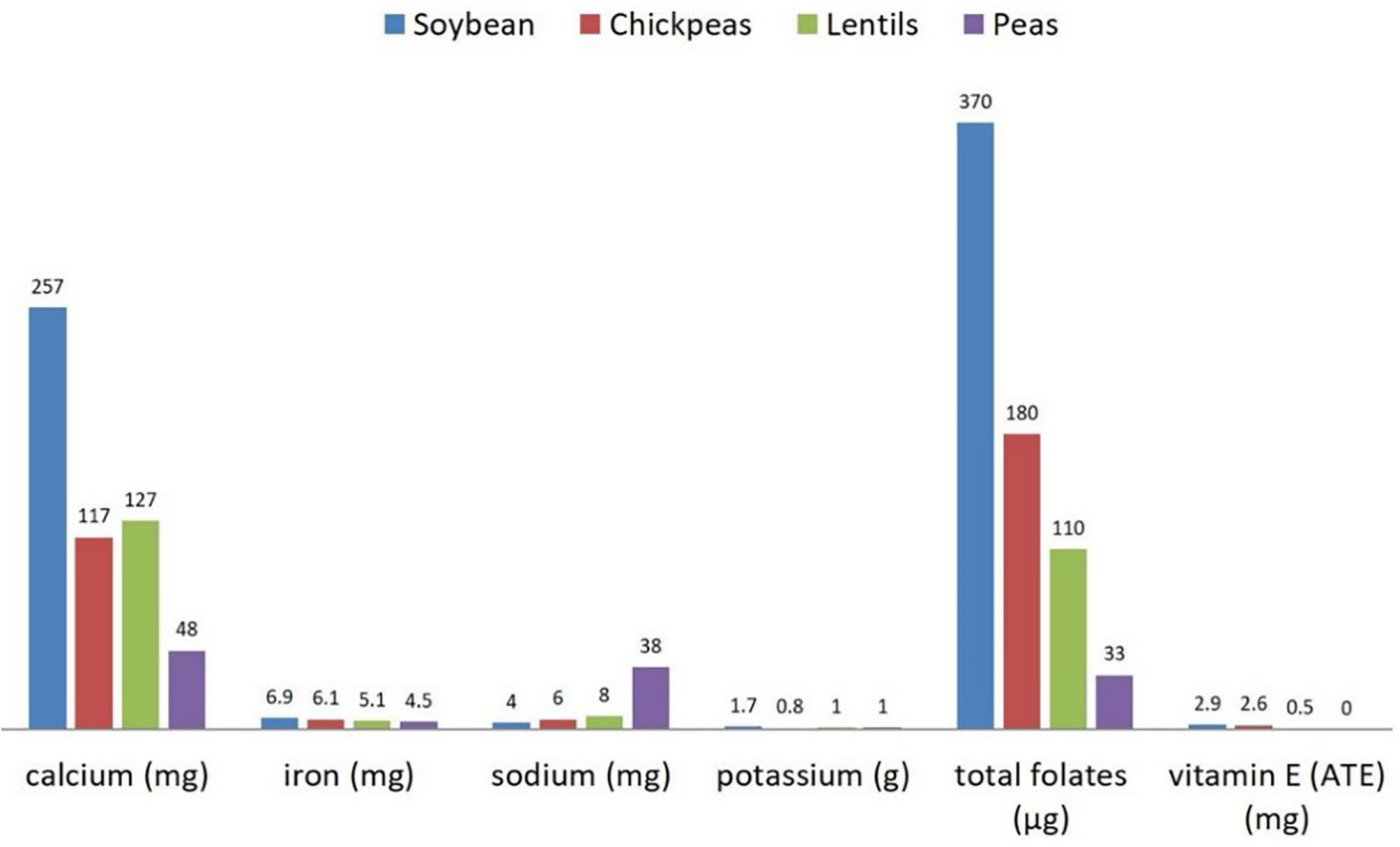
Micronutrients' content (per 100 g) in raw dry soybeans, peas, chickpeas, and lentils. Source: reference (8).

Understanding the nutritional composition of soybean in terms of macronutrients, micronutrients, and many minor bioactive compounds is crucial to evaluate the nutritional properties and limits (Table 1, Figures 1, 2).

**Table 1:** Main constituents of soybean, mature seeds, and raw dry soybeans.

	**Nutritional value for 100 g**	**Comments**
Energy	1,866 kJ (446 kcal)	
Protein	36.49 g	
Essential amino acids:		Soy protein, although plant-derived, is a complete protein; it contains a great quantitative of lysine, while methionine is a limiting amino acid
Histidine	1.097 g	
Isoleucine	1.971 g	
Leucine	3.309 g	
Lysine	2.706 g	
Methionine	0.547 g	
Phenylalanine	2.122 g	
Threonine	1.766 g	
Tryptophan	0.591 g	
Valine	2.029 g	
**Other proteins:**		
**Enzymes**		
Lipo-oxygenase		The unpleasant bean flavor typical of soybeans is due to the content of lipo-oxygenase
Urease		Urease is present in uncooked soybean and is progressively destroyed by heat, so it can be used as an indicator of adequate heat treatment
**Antinutritional factors:**		
Soy protease inhibitors		Soy protease inhibitors reduce protein digestibility
Lectins		Lectins can interfere with micronutrient absorption
Fats^a^	19.94 g	The fat content consists mainly of unsaturated fatty acids, with smaller amounts of saturated fatty acids and no cholesterol
Saturated	2.884 g	
Monounsaturated	4.404 g	Soy is high in polyunsaturated fatty acids, which are essential fatty acids with a hypocholesterolemic effect
Polyunsaturated^b^	11.255 g	Omega-3 ALA 0.72–2.16% Omega-6 LA 6.48–11.6%
Carbohydrates	30.16 g	
Sugars	7.33 g	Soluble sugars increase palatability, thus potentially causing flatulence
Dietary fiber	9.3 g	Dietary fiber is a non-digestible portion of food. In soybean, it is composed of lignin, enzyme-resistant starch and oligosaccharides, and cell wall polysaccharides (cellulose, hemicellulose, and pectins)
Minerals^c^		Although soybean's mineral content is high, bioavailability is slow since phytate reduces the absorption of dietary minerals, particularly for zinc and iron
Potassium	1.797 mg	
Calcium	277 mg	
Magnesium	280 mg	
Copper	1.658 mg	
Iron	15.7 mg	
Zinc	4.89 mg	
**Other constituents**		
Phytic acid		Phytic acid impairs mineral absorption and may promote mineral deficiencies
Isoflavone^d^		Plant compounds have lower estrogenic activity than the human female hormone 17-β-estradiol and other estrogenic receptor-independent effects, which can play a role in normal sexual development and reproductive function

*Table 1 source: Reference (3)*.

a *Source: reference (4)*.

b *Source: reference (5)*.

c *Source: reference (6)*.

d *Source: reference (7)*.

*ALA, alpha-linolenic acid; LA, linoleic acid*.

### Proteins

Soybeans contain a high quantity of proteins of up to ~40–41% of their dry weight. About the 65–80% of this amount is represented by storage proteins. Among these, glycinin and β-conglycinin are the major ones, the former being richer in sulfur-containing amino acids (methionine and cysteine) than the latter. The remaining proteins are mainly used by the seed itself to mobilize stored nutrients and defend it against microorganisms/macroorganisms, to assure proper growth (2).

#### Proteins: Nutritional Quality

Since 2013, in order to assess the protein quality, the World Health Organization/Food and Agriculture Organization (WHO/FAO) adopted an evaluation methodology based on protein digestibility (increased by heating and fermenting soy) and the ability to provide an adequate amount of indispensable amino acid to meet organism requirement [digestible indispensable amino acid scores (DIAAS)] (9, 10).

As previously mentioned, purified or concentrated vegetable proteins have high digestibility (>95%), while for some intact vegetable products, protein digestibility is lower (around 80–90%). Moreover, one of the most used methods by agencies around the world to evaluate protein's nutritional quality is the protein digestibility-corrected amino acid score (PDCAAS). This rating measures the quality of specific food protein comparing against a standard food protein based on the amino acid requirements of a preschooler adjusted for digestibility. Isolated soy protein has a PDCAAS of 1.0, which is the highest value that any protein can achieve and the same score as milk protein and egg white (11, 12).

The high nutritional quality of soybean protein is reaffirmed by the fact that it is high in lysine, and therefore, it may be a valuable supplement to cereal foods, which, conversely, have a low content of lysine (1).

#### Proteins: Enzymes and Antinutritional Factors

A small part of soybean proteins is represented by soybean protease inhibitors. Among these, the best known are the trypsin–chymotrypsin inhibitor and trypsin inhibitor (13). Various studies have demonstrated how these proteins can inhibit pancreatic enzymes, thus reducing the digestibility of proteins (1, 2). Among these antinutritional factors, lectins also deserve to be mentioned as they interfere with the absorption of micronutrients. Soybean protease inhibitors and lectins are both inactivated by heat treatment and fermentation (2, 9). As for enzymes, lipo-oxygenases are not antinutritional factors but give the soybean their characteristic, undesirable, beany flavor (14, 15), whereas urease is used mainly in production processes as an indicator of adequate heat treatment (1, 16).

### Lipids

Soy contains various types of lipids; among these, the most numerous are triglycerides (96%). Smaller percentages are represented by phospholipids (2%), unsaponifiable lipids (1.6%), free fatty acids (0.5%), and traces of carotenoid pigments (1). The lipid content of soy is important because soy oil is rich in polyunsaturated fatty acids (PUFAs), linoleic and linolenic acids, which are essential fatty acids (EFAs) that cannot be synthesized by mammals and therefore need to be obtained from the diet (2). These EFAs are metabolized in long-chain PUFAs (LCPUFAs), in particular, linoleic acid (LA; C18:2 *n*−6), which is the progenitor of *n*−6 long-chain LCPUFA series [arachidonic acid (AA), C20:4 *n*−6], while alpha-linolenic acid (ALA; C18:3 *n*−3) is the progenitor of *n*−3 LCPUFA series eicosapentaenoic acid (EPA; C20:5 *n*−3) and docosahexaenoic acid (DHA; C22:6 *n*−3). It is now known that PUFAs provide important health functions, especially in cardiovascular health, mainly by improving plasma lipid profile and thereby lowering cardiovascular risk (17, 18). Notably, LA dietary intake is inversely correlated with cardiovascular risk by modulating several cardiometabolic factors, such as the cholesterol-lowering effect and beneficial effect on glucose metabolism (6).

### Carbohydrates

In terms of composition, the second macronutrients more represented are carbohydrates, whose percentage is around 35% of dry seed weight (a mature soybean seed contains approximately 40% of proteins, 20% of lipids, 35% of carbohydrates, and 5% of minerals of dry weight).

Soy contains two groups of carbohydrates: soluble sugars (sucrose 5%, stachyose 4%, and raffinose 1%) and insoluble fibers (20%), which are structural carbohydrates. Sucrose is an important carbohydrate because it makes soy more palatable, giving it a very sweet flavor (2, 19). Raffinose and stachyose are unwelcome components in soybean seeds because they are the major cause of soybean flatulence, a challenging problem associated with the consumption of soybeans. Since humans do not have enzymes capable of processing them, they reach the intestine undigested. At that level, being non-nutritional factors, they are fermented by intestinal flora bacteria, which, as a consequence, produce gas and flatulence (1, 2, 19).

### Minerals

Minerals represent 5% of dry seed weight. The main constituents are potassium, calcium, and magnesium, while others, such as iron, zinc, and copper, are present just in traces, thus requiring supplementation (1).

### Other Constituents

Two other important constituents of soybeans are phytic acid and isoflavones. Phytic acid is found in soybeans as phytin salts, which are not available to humans and can chelate a series of micronutrients among which especially zinc, calcium, magnesium, and iron, thus preventing their mucosal absorption (20). Therefore, it is considered an important antinutritional factor. Soy is also rich in isoflavones (daidzein, genistein, and glycitein), also known as phytoestrogens since their structure is similar to that of estrogens. They are capable of binding estrogen receptors (ERs), although less strongly, increasing circulating estrogens levels. Current evidence on a possible link between isoflavone consumption and various diseases remains dubious, especially in terms of their capacity to prevent cardiovascular pathologies and osteoporosis and to promote cancers (2).

## Role of Soy in Infant Feeding

Various factors contributed to the extensive use of soy-based formulas and products among children. Among others, the most important is the high nutritional value and palatability (21). Soy-based formulas were first introduced to the western market almost 100 years ago. In particular, their appearance in the USA dates back to 1909 when they were proposed as an alternative for feeding children with an allergy to cow's milk proteins (CMA) (22). At the time of their first introduction, soy-based formulas were made from soy flour. Soon, however, various limiting factors such as low digestibility and high quantity of fibers and phytates induced the market to find alternative solutions and led to the development of soy-based formulas containing proteins isolated from soy (soy protein isolate) (23, 24). Soy protein isolate is extracted from the flake using different solvents to obtain a final proteic source with higher digestibility and nutritional value since it contains higher amounts of essential amino acids (23).

Throughout history, soy-based formulas have undergone various rearrangements. In particular, in the 1970s, amino acids such as methionine (an essential amino acid), taurine, and carnitine, which are poorly contained in soy protein, were added to formulas (23). In 2000, soy-based formulas also started being supplemented with LCPUFAs (24).

### Soy-Based Infant Formula Indications and Nutritional Safety

Soy-based formulas currently on the market do not contain cow's milk proteins and lactose (25). Therefore, the main current indications for their use in infancy are allergy/intolerance to cow's milk-based formulas (CMF), hereditary lactase deficiency, and galactosemia (23, 25). Lactose-free formulas can be considered in the management of acute gastroenteritis in hospitalized children age <5 years, but their routine use is not recommended in an outpatient setting (26).

#### Soy-Based Infant Formula Nutritional Composition

The nutritional composition of formulas based on soy protein isolates, as compared with human milk (HM) and cow's milk, is shown in Table 2. The protein source is represented by soy protein isolates with supplementations in methionine, carnitine, and taurine. The fat content is made up mainly by vegetable oils, in particular soy, palm, sunflower, olein, safflower, and coconut oil. Currently, DHA and AA are added to all formulas based on soy protein isolates (25). Phosphorus and calcium are present at concentrations 20% higher than those present in CMF. Currently, soy-based formulas undergo a heating process to eliminate protease inhibitors, thus increasing digestibility. This heating process neutralizes up to 90% of protease inhibitors. Another issue with soy-based formulas is the high quantity of phytates and fibers, which can bind zinc and iron. Over time, this issue has been resolved by fortifying them with iron and zinc (23, 25).

**Table 2:** Nutritional composition of human milk (HM), cow's milk (CM), soy-based beverages (SB), soy-based infant formula as regulated by EU (SIF-EU), and Italian commercialized soy-based formula (SF).

	**HM^a^**	**CM^a^**	**SB^b^**	**SIF-EU^c^**	**SF^d^**
**COMPOSITION IN 100 g**					
Energy (kcal)	70	62	32	60–70	67–68
Water (g)	87.5	87.7	89.7		
Total protein (g)	1.0	3.3	2.9	1.35–1.96	1.6–1.7
Total fat (g)	4.4	3.3	1.9	2.64–4.2	3.3–3.5
Lactose	6.9	4.7			
**MINERALS**					
Calcium (mg)	32	112	13	30–98	55–68
Iron (mg)		0.1	0.4	0.18–0.91	0.9–1.1
Magnesium (mg)	3	11		3–10.5	
Phosphorus (mg)	14	91		15–63	39–40
Potassium (mg)	51	145	120	48–112	68–77
Sodium (mg)	17	42	32	15–42	20–27
Zinc (mg)	0.2	0.4	0.2	0.3–0.7	
Copper (μg)	100			36–70	
Selenium (μg)	1.8	1.8		1.8–6.02	
Manganese (μg)		8		−70	

a *Source: reference (27)*.

b *Source: reference (28)*.

c *Source: reference (29)*.

d *Source: reference (30)*.

#### Nutritional Safety of Soy-Based Infant Formula

Given the many pros and cons of soy, the health effects of soy-based formulas have been intensely investigated over the last few years, primarily for their high phytoestrogen content and, thus, their impact on children's health and chronic disease risk (31).

A systematic review and meta-analysis by Vandenplas et al. (23) evaluated the safety of soy-based formulas in chronic administration, analyzing cross-sectional, case-control, cohort studies, and clinical trials published until 2013 in which the sexual development of SF formula-fed infants was compared with that of children fed with other formulas. The authors concluded that SF is a safe alternative to CMF, given that, although some differences could be detected, such as values of genistein and daidzein, which were higher in children fed SIF than in children fed CMF or HM, the long-term effects of isoflavones on the main reproductive functions in humans were clinically irrelevant. In particular, anthropometric patterns, bone mineral content, and neurological, reproductive, immune, and endocrinological outcomes in SF formula-fed infants were similar to those in CMF-fed infants (32–34). According to these data, a recent review of Messina et al. (31) shows that soy can be safely incorporated into the children's diet, always following the principles of variety and moderation. There is little evidence that soy exerts adverse hormonal effects in children, but data are very limited.

Some studies show that soy consumption in early childhood can be related to altered thyroid function, although there are very few studies in children, and most of the conclusions are based on old case reports, published before the introduction of iodine-supplemented SF (25, 35, 36). A retrospective study of Fort et al. (37) evaluated the prevalence of autoimmune thyroid disease in breast-fed and SF-fed children, obtaining a history of feeding practices in 59 children with autoimmune thyroid disease, their 76 healthy siblings, and 54 healthy non-related control children. The frequency of feedings with SF in early life was significantly higher in children with autoimmune thyroid disease (prevalence 31%) than in their siblings (prevalence 12%) and healthy non-related control children (prevalence 13%). In a more recent study, Conrad et al. (38) analyzed the effect of childhood diet on thyroid function in children with congenital hypothyroidism, testing the hypothesis that SF-based feeding in infants with congenital hypothyroidism leads to a prolonged increase of thyroid-stimulating hormone (TSH). They evaluated eight children in the soy diet group and 70 in the non-soy diet group, finding that SF-fed children had a prolonged increase of TSH than had children fed with non-soy formula. As a result, not all the issues regarding the safety of SF in infants have been solved.

This field is still highly controversial because it is still difficult to assess whether soyfood consumption early in life (<24 months of life) is completely safe with no hormonal effects at all; thus, further research is needed to establish that SF given early in life is safe and beneficial from a nutritional point of view.

## Soy-Protein Formulas and Cow's Milk Allergy

Soy-protein formulas are widely used for feeding children with CMA. Cow's milk allergy is an adverse immune response associated with a clinical reaction due to the binding of specific immunoglobulin (IgE) to antigens/proteins in cow's milk (IgE), which could induce an allergic reaction in sensitized individuals (39). It is the most common type of food allergy, affecting <2% of children under 4 years of age (40).

The general treatment for CMA consists of avoiding exposure to the implicated allergens: patients must avoid cow's milk and cow's milk protein-based products. If breastfeeding is not feasible, replacement with a substitute appropriate formula is mandatory (41). Extensively hydrolyzed formulas (eHFs), in which milk proteins have been fragmented (hydrolyzation) to make them less allergenic, are the first choice in children diagnosed with CMA. They are nutritionally adequate and well-tolerated by children allergic to cow's milk proteins, although among their main drawbacks is poor palatability, high costs, and the remote potential to cause anaphylaxis. From this point of view, amino-acid formulas (AAF) are safe but very expensive, unpalatable, and not widely available (42). Therefore, the choice for a different milk formula increasingly falls on soy formulas (SF) and rice-hydrolyzed formulas (RHFs), which are well-tolerated and considered a second-line resource in infants with IgE-mediated CMA (43).

In the last few decades, soy formulas have been changed over the years to improve digestibility, nutritional values, and protein quality. Contemporary soy formulas are supplemented with appropriate amounts of amino acids such as methionine, taurine, and carnitine, and they are not deficient in zinc, calcium, or phosphorus. Nevertheless, despite all these benefits, two potential drawbacks remain for the use of soy-based formulas in infants with CMA.

One important concern regard phytoestrogens in the form of isoflavones present in soy protein and their possible hormonal effects on the reproductive system. Indeed, phytoestrogens are plant compounds with structure and function similar to those of 17-β-estradiol that, thanks to this particular chemical structure, are able to recognize and to bind ERs and thus exert an agonistic or antagonistic effect on this hormone. These chemical agents perform an estrogenic action either directly (binding the ERs, enhancing aromatase activity, and increasing sensitivity to estrogens) or indirectly, through their effect on GnRH, thus leading to an increase in endogenous production of estrogens. They are present in a very large amount in soy-based foods, although their stimulus on the receptor is 100–1,000 times less than the human female hormone 17-β-estradiol.

Over 94% of the phytoestrogens contained in SF are present as beta-glycosylated isoflavones, such as genistein, daidzein, and glycitein, which are biologically inactive and very poorly absorbed when in this form (44). Their activation occurs in the gut, even if the number of active compounds that are absorbed and enter in the blood varies from subject to subject, primarily according to the composition of the gut microbiota. Moreover, the liver plays a central role in determining the concentration of active isoflavones in the blood because a large portion of them are metabolized, thus reducing the number of compounds that can exert an estrogen-like activity (45). However, phytoestrogens have other ER-independent effects, such as the alteration of epigenetic marks and the inhibition of estradiol, which can play a role in the development of early puberty in females and sexual disorders in males (7).

Although data are limited since relatively few studies have been conducted in children, some authors currently support the possible correlation between phytoestrogens and the development of permanent modifications of reproductive system function (46, 47). A Korean case-control study involving 150 girls found that genistein blood concentration in girls with precocious puberty was significantly higher than in the control group (48). In contrast with these findings, most of the studies about soy and pubertal development show no conclusive evidence that SF has hormonal negative effects on children or affect pubertal development, as is confirmed in a systematic review with meta-analysis by Vandenplas et al. (23). Sinai et al. also confirmed this by conducting the first prospective, physical examination-based study, demonstrating no association between SIF consumption and growth and puberty parameters (49). Given that data are conflicting on these points, as a precaution, the European Society for Pediatric Gastroenterology, Hepatology, and Nutrition (ESPGHAN) recommends that children under 6 months of life should not be fed with soy milk as their only source of nutrition (31, 50).

The other concern about SF is the use of transgenic soybeans. Although available data suggest no deleterious effects on the human genome, concerns about the use of transgenic food persist (51). Since soy has a long history of successful use in managing CMA in infants, to better predict the usefulness of soy proteins for controlling food allergies, it is important to understand the relative allergenic reactivity of soy compared with other major food. The antigenicity of soy has been suspected since 1934 (52) and documented in 1982 by Eastham et al. (53). Several studies have measured the proportion of infants with documented CMA that developed soy allergy when SF was used as a replacement for cow's milk formula. Zeiger and Sampson (54) evaluated 93 children <3.5 years of age with documented IgE-associated CMA by double-blind, placebo-controlled food challenge: soy allergy occurs in 14% of children with IgE-associated CMA, whereas Businco et al. (55) implicated soy allergy in 4% of 143 children. Bock and Adkins (56) reported that 7% of CMA infants developed soy allergy when switched to SF. In the study by Klemola et al., 10% of CMA infants developed SF allergy, and adverse reactions to soy occurred more frequently in children under 6 months of age than those of 6–12 months (5 out of 20 vs. 3 out of 60) (57). These studies show that SF can be a safe alternative to cow's milk in children with IgE-associated CMA and reported that infants allergic to cow's milk could be effectively managed with SF if soy tolerance is proved at the time of introduction of SF.

This overall performance approaches the clinical standard for hypoallergenic formula, where a hypoallergenic formula is a formula that meets the criterion characterized by a clinical tolerance of 90% in children with certain CMA (95% confidence that 90% of allergic infants will not react) (58). Nevertheless, given all these pros and cons, the ESPGHAN recommends not using soy in infants with food allergy during the first 6 months of life and not in preterm infants (50). For the management of CMA, it is preferable to use eHFs of cow's milk proteins in the first instance (59). Growth patterns, bone health, and metabolic, reproductive, endocrinological, immune, and neurological outcomes of SF-fed infants are similar to those observed in children fed with CMF (23). Therefore, after the first 6 months of life, SFs may be considered if tolerance to soy proteins is established, and SF can be suggested as a first-choice alternative for infants >6 months of age with CMA.

### Could Soy-Protein Formulas Be Used for the Prevention of Cow's Milk Allergy?

Prevention of allergic diseases in high-risk children is a difficult challenge, and at the moment, only breastfeeding is suggested as useful in these patients; if breastfeeding is not possible, the recommended approach is a diet with CM eHF, although rice or soy infant formula can be considered as a second option (60, 61). A study on SFs used for the prevention of CMA published in 1953 reported that SFs prevented the onset of a food allergy if given to atopic children (62), although the results were criticized mainly because of the lack of a control group. Among other studies published later, some have concluded recommending the use of SFs because subjects showed fewer allergic reactions than did infants fed with cow's milk formulas, while others found a similar frequency of allergic manifestations with both formulas. Bardare et al. (63) confirmed the preventive effect of SFs in a large prospective study including 391 atopic infants. At the end of the first step (1 year of life), 13% of the participants in the study group and 29% of the participants in the control group had an atopic disease.

On the contrary, a meta-analysis by Osborne et al. published in 2004 showed contradictory results: feeding with an SF should not be recommended for the prevention of allergy or food intolerance in infants at high risk for these diseases (64).

The argument is still greatly controversial, mainly because most of the studies that have been conducted either lacked scientific criteria for diagnosing soy allergy or misinterpreted the conclusions. Thus, further studies are necessary to study not only the prevalence of soy allergy in children with CMA and the entire pediatric population but also the preventive effect of soybean on allergic disease development. In addition, we suggest planning double-blind placebo-controlled oral food challenge studies in larger cohorts of children to compare the efficacy and safety of SFs in children with IgE-mediated CMA and to determine further ways to prevent CMA in atopic and allergic children.

## Use of Soybean and its Derivates in Plant-Based Diets

One of the main factors that has influenced the popularity of soy foods is the ever-growing interest of the population toward plant-based diets and their many health benefits, together with the recognized high protein quality of soy (31, 65–67).

### Plant-Based Diets

The term “plant-based diets” includes many different dietary patterns that comprise higher quotas of plant products (vegetables, fruits, whole grains, legumes, nuts, and seeds) than animal ones (66). Specifically, there is evidence that soy foods may reduce low-density lipoprotein (LDL)-cholesterol levels and modestly lower blood pressure, thus reducing the risk of coronary heart disease (68). Nevertheless, further research is required before unequivocal conclusions can be made. By definition, a vegetarian diet is one that excludes the consumption of meat and fish. Its most common variant is lacto-ovo-vegetarianism, which allows the consumption of eggs, milk, and cheese. Conversely, the most restrictive variant of plant-based diet is veganism, which prohibits all animal-derived food (65, 69, 70).

It is important to emphasize that the health benefits gained from adhering to a vegetarian or vegan diet can be obtained also through an omnivorous diet, involving the reduction of meat and an increase in the intake of plant foods (66, 69). Together with the well-known health benefits, plant-based diets are associated with less environmental impact. However, a sustainable diet can also be achieved with dietary patterns characterized by low to moderate amounts of animal-based food and a high intake of plant-based food, such as the Mediterranean diet (66).

In Italy, the percentage of people following a vegetarian diet (vegan or lacto-ovo-vegetarian) has doubled in the past 5 years and is around 7.3% (71). Although it is not possible to know the exact number of vegetarian children, vegetarian parents frequently choose vegetarian diets for their children (72).

### Plant-Based Diets and Nutritional Requirements for Children

Well-planned plant-based diets can provide adequate nutrition requirements throughout all stages of life. Nevertheless, special attention should be paid to children following a vegetarian or vegan diet, especially during complementary feeding (73, 74). Indeed, although theoretically a vegan diet can meet nutritional requirements when medical and dietary advice regarding supplementations is followed, the risk of severe deficiency, especially for vitamin B12, is present. Therefore, if parents choose a vegan diet for the complementary feeding of their infant, regular medical and dietetic supervision is necessary to avoid the risk of irreversible cognitive damage.

Diets derived from the VegPlate Junior (VPJ) method, based on six foods including grains, protein-rich foods, vegetables, fruits, nuts and seeds, and fats, have recently been considered well-planned plant-based diets (75). Regarding protein-rich foods, since soy and its derivatives have essential amino acids in a proportion similar to those in animal foods, their consumption should be encouraged.

Another criterion to define a plant-based diet as well-planned is to consume a wide variety of plant foods (73). Being plant food sources of protein very variable in terms of digestibility, quality, and composition of essential amino acids, to reach adequate protein requirement, it is important to combine different plant foods to provide all the essential amino acids. In the case of soy, for example, since methionine is an essential limiting amino acid, it would be optimal to combine it with cereals that are rich in methionine but lack lysine.

The risk of nutritional deficiencies in plant-based diets increases the more restrictive the diet is and the younger the child (69). In the first year of life, the infant can be fed either breast milk or formula milk, and at this age, the only alternative to cow's milk formulas are plant-based formulas such as soy-based formula (72).

### Soy-Based Beverages and Foods

Despite being called soy milk on the market, it is important to highlight that soy beverages are not nutritionally comparable with milk (69) (Table 2). Regarding this issue in Europe, to fight misleading labels, Council Regulation 1234/2007 prohibits the use of the word “milk” for drinks that are not made from mammary secretion (76).

Since they can cause severe nutritional deficit (77), these drinks should not be chosen as a cow's milk alternative in children younger than 24 months (78). Indeed, soy beverages have lower energetic levels and contain fewer carbohydrates, fats, calcium, and vitamin B12, than have cow's milk. However, compared with CM, they are a good source of trans fats, monounsaturated fatty acids (MUFAs), and PUFAs (ALA and LA). In addition to the above-mentioned advantages, soy drinks have a higher protein quality than other plant-based drinks; furthermore, they are lactose and cholesterol free (79).

Soy may have a role in childhood nutrition also during complementary feeding, such as tofu, which is obtained by curdling the liquid extracted from pressed soybeans. Many vegetarian or Asian mothers choose to use tofu because it has a soft consistency that makes it easy to blend with other cereals, has great palatability, and is rich in calcium, which makes it nutritionally adequate for a baby or a toddler (21).

## Soy-Based Formula and Preterm Infants

Although the soy-based formula is considered safe for healthy and full-term infants, both the American Academy of Pediatrics (AAP) and the ESPGHAN do not recommend its use in preterm infants (25, 50, 80).

### Aluminum Content and Risk of Osteopenia in Preterm Infants

Taking into account that aluminum competes with calcium absorption, preceding studies have reported that the consumption of soy-based formulas by preterm infants could increase the risk of osteopenia due to their higher aluminum content as compared with cow's milk-based formula and breast milk (17, 18, 24, 81). To solve this issue, modern soy-based formulas have been supplemented with high amounts of phosphorus and calcium, while the aluminum content has been maintained lower than the tolerable intake, according to the Joint WHO/FAO Expert Committee on Food Additives (21, 82). However, the long-term effects of early aluminum exposure have not been fully elucidated yet (23, 25). In a random, controlled study of very low birth weight (VLBW) infants from 3 to 8 weeks of age, Hall et al. (83) reported that VLBW infants randomized to be soy-based formula-fed supplemented with calcium, phosphorus, and vitamin D, showed a lower weight gain and serum protein concentration than those randomized to be whey-predominant premature infant formula-fed. In light of the specific high nutritional requirements and vulnerability of preterm infants, further insights are needed before their use can be proposed in preterm infants (12).

## Key Messages

Modern soy-based formulas are adequate to ensure normal growth patterns in healthy infants, but they have few indications as a substitute for cow's milk formulas: allergy or intolerance to cow's milk, galactosemia, severe persistent lactose intolerance, post-infection diarrhea, and vegan diet.

Formulations have changed over the years to improve digestibility, nutritional values, availability of minerals, and protein quality, and the data suggest that modern soy-based formulas are well-tolerated and support normal growth patterns and nutritional status in healthy term infants.

In infants with cow's milk allergy, soy-based formulas may be considered as a first choice alternative to CMF only after 6 months of life, if tolerance to soy protein is established, although soy-based drinks should not be used as a substitute for cow's milk in children <24 months old.

The ESPGHAN and AAP recommend not using soy in infants with food allergy during the first 6 months of life and not using soy in preterm infants.

It is still unclear if the routine use of soy-based formulas may have roles in the prevention of allergic diseases, and further large-scale studies are required to clarify the safety of soy and its use for the treatment of cow's milk allergy.

## Conclusions

Since their first use as cow's milk substitute formulas for children with CMA, many changes have occurred over the years to improve digestibility, nutritional values, and protein quality of soy-based formulas.

Modern soy-based formulas appear to be adequate to ensure normal growth patterns and development in healthy infants, although they do not appear to have nutritional advantages over cow's milk formulas. Indeed, in term infants, although isolated soy-protein based formulas may be used to provide nutrition, there are few indications for their use as a substitute of cow's milk formulas: indications include allergy/intolerance to cow's milk, galactosemia, severe persistent lactose intolerance, post-infection diarrhea, or vegan diet. Beyond these, there are no valid indications for replacing cow's milk with soy-based drinks, also remembering that, up to 6 months of life, HM remains the best way to feed infants. It is still unclear if soy protein formulas may have roles in the prevention of allergic diseases, but, for CMA infants, they certainly represent a valid, economic, and well-tolerated alternative to eHFs.

Worries are related to the use of soy in children regarding phytoestrogens and the use of transgenic soy, with their possible hormonal effects on the reproductive system, neurodevelopment, obesity, and gut microbiota development. There is no conclusive evidence that soy isoflavones can adversely affect development, especially the reproductive system and endocrine function, although there are little data available on the potential effects of phytoestrogens in young children on subsequent sexual and reproductive development. Similar considerations hold for use of transgenic soy: the available data suggest no deleterious effects on the human genome. Concerning growth patterns, clinical studies have shown that during the first years of life, there are no significant differences between infants fed SF and those fed cow's milk formulas. As the matter is still debated, the ESPGHAN and AAP recommend not using soy in infants with food allergy during the first 6 months of life and not using soy formulas in preterm infants. After 6 months of life, SFs may be considered if tolerance to soy proteins is established and SF can be recommended as a first-choice replacement for infants >6 months of age with cow's milk allergy.

Although significant advancements have been made in recent years in our understanding of soy properties, substantial gaps in our knowledge still exist; for many reasons, it is still difficult to establish whether soy-based food consumption early in life is safe and beneficial; thus, we recommend that soy drinks should not be used as a substitute for infant formulas or cow's milk in children younger than 24 months. Further additional studies will be needed to clarify the effects of soy on the reproductive system, long-term effects on neurodevelopment, the effects of glyphosate, effects on the microbiome, and, generally, all the long-term consequences of soy.

## Author Contributions

EV, LC, GN, and DP made a substantial contribution to conception, design, and acquisition of data. EV, LC, GN, LR, GV, ED'A, MG, GZ, and DP drafted the manuscript and critically reviewed it for important intellectual content. All authors gave the final approval of the version to be published.

## Conflict of Interest

The authors declare that the research was conducted in the absence of any commercial or financial relationships that could be construed as a potential conflict of interest.
